# Sister Olive: A Hospital Romance

**Published:** 1887-01-01

**Authors:** Walter J. Eccott


### SISTER OLIVE: A HOSPITAL ROMANCE.

By WALTER J. ECCOTT.

CHAPTER VI.

Very soon Sister Olive returned, and Grant went down to Mrs. Tenison. She was anxiously awaiting him at the foot of the stairs, and led him into the dining-room where supper was laid. After assiduously supplying his wants with refreshment, of which he stood in need from his prolonged exhaustive mental excitement, she sat awhile in silence. But her face asked the questions she hesitated to frame in words. Grant read them easily there, and soon began:

"You are no doubt anxious to hear what I think of your son, Mrs. Tenison, and I'm sorry I cannot speak in any way definitely. Cases like his are of rare occurrence. It may be very serious. But it has not reached the most critical stage. Perhaps we may prevent the fever reaching a dangerous stage at all. He is in, as regards nursing, the best possible hands. Not a single effort will be lost, and in cases like his, nursing is the great thing. As far as my medical experience goes, I need not say it will be freely devoted. And if to-morrow morning I see any necessity for it,—for then I shall be better able to judge,—I will get Professor Temston to come up. Archie has taken a sleeping-draught now, and will get some hours' rest to-night. I should advise you to go up and say good-night to him, and then go to bed. You look tired and harassed. Leave Archie to us with complete confidence. Your sitting up would be of no use, and only make you feel worn out the next day. Let one of the servants stay up in the little room upstairs."

"Thank you very much," said Mrs. Tenison, gratefully. "It's very good of you to stay. I'll show you your room—you've taken a great part of the load off my mind. I must come up with you and say good-night to my boy; and if there is anything I can do before I go to bed, please tell me and I will see to it myself."

Grant gave a few directions, and then followed Mrs. Tenison upstairs. She bent over her boy, who was not yet asleep, and kissed him fervently, scarcely being able to murmur, "Good-night, Archie! You'll surely be better in the morning!" Then she reluctantly turned away, and, her eyes looking wistful and glistening with the tears that were ready to fall, she bestowed a motherly kiss and a whispered "God bless you, Sister Olive," on that loving minister to sickness. Olive twined her arm in the sore-hearted mother's and left the room with her. And when she returned, presently, she looked as if she too had been deeply affected by her "Good-night" to the anxious mother, whose heart was overflowing with an inexpressible yearning to save her boy.

For some time Archie tossed restlessly, breathing quickly, and complaining of aches in his back and limbs: every now and again, too, of thirst. With tender solicitude Sister Olive held a cooling draught to his lips. Little by little he became more listless, his face more pinched and dejected. At last the sleeping-draught worked potently, and he fell asleep.

Before retiring for the night, Grant again visited the patient's room and took the temperature. It had risen considerably since he first came. The skin was very hot and dry. The fever was evidently raging. The doctor's face grew serious.

"What do you think of him now?" asked Sister Olive. "Is it very dangerous?"

"I am afraid so," returned Grant slowly; "but we shall see how he is after this sleep. That will last some hours. Meanwhile, as there is no object in my sitting up, and as I shall want all my wits about me to-morrow, I shall go to bed."

"Yes! go, by all means!" said Olive. "You look tired out. Besides, he's not likely to wake for some hours. But when he wakes?"

"Oh, call me at once!" said Grant, "and then your watch will be relieved." For a while no sound was heard but the breathing of the patient. As he turned to leave the room, he said in a low voice:

"I think you are interested in the case of John Hargen?"

"Yes!" said Sister Olive eagerly. "How is he?"

"Much better! I've had him removed to a cottage in the Fothercliffe Road, the last house in the town—Petunia Cottage: a Mrs. Hussey is the name—where he'll be very comfortable and pick up his strength in time. Pure air, good food, and a good-natured landlady, three prime requisites, he has."

Her face brightened as she said: "I'm very glad, very glad. And it has been very good of you, Dr. Grant, to take such interest in a chance infirmary-patient."

"I don't think I can take any credit," he said. "At first it was a purely medical interest I took. Then there was an added incitement, because I somehow fancied that you took more than an ordinary interest in it: and later, because, from some words Hargen let fall, I guessed that his history was in some way bound up with your own."

Sister Olive looked startled, and indeed almost pained. She sighed slightly as she said:

"Yes! he is. And it was a strange chance brought him into the Infirmary. Did he?" she faltered—"did he tell you anything?"

"Yes, Sister Olive. To-night I have heard from his lips, spoken of his own accord, the story of his life. I did not ask for it. I had no idea of what was to be unfolded. To-night I know what burden you would rather have borne yourself in silence than share it with another, lest it weighed him down. But if I can read John Hargen now, this illness has been the crisis of his life. He will go forth into the world a wiser and stronger man. At last he feels, with the bitterest regrets that he did not sooner recognise what you have been to him."

"Please do not talk more of it now, Dr. Grant!" she pleaded, evidently troubled. "As it has happened thus by accident, there is little to be said. Perhaps it has been providential. Now you have had a glimpse into the past, you know more of me, and, whether my course has been Quixotic or not, I could not do otherwise.

Say no more now. We two have work in hand here. Let us do that. Then there will be time for talking. Good-night, Dr. Grant." And with a silent pressure of the hand, Dr. Grant left the sick-room and sought his own.

It was about six the next morning when Archie awoke. He was conscious, but very tired and listless. At the first stir Sister Olive was at his side, and asked, in her softest tones:

"Well, Archie, how do you feel now?"

"Headache!" he gasped. "Please—give—thermometer. Where's Grant?—I'm thirsty." He spoke with difficulty.

She skilfully raised his head a little and administered some spoonfuls of beef-tea. He seemed to take it as quite a natural thing to be treated as a child, and sank down again in the bed.

Presently Grant came in, and bade Sister Olive go and take her needed rest. She whispered what Archie had said, and left. Archie strained his eyes after her, and then seemed to take little notice of anything. He asked no question of Dr. Grant as the latter proceeded to examine him. The skin had become considerably distended the whole length of the arm. There were evident signs that the poisonous matter had gathered and spread. For this reason he sent off a messenger for Professor Temston, whose reputation as a surgeon was unequalled in that part of England, rather than take further responsibility himself.

In about an hour and a half the Professor came. The fever had by this time increased to delirium. He agreed with Dr. Grant that the time had come for more active measures. Skilfully and rapidly he did what was necessary, and, after a long consultation with Dr. Grant, left the case in his hands.

Some time after, having given directions of the course to be pursued, and of where he was to be found if anything untoward should occur, and with a promise to call at mid-day, Dr. Grant returned to Cottingham.

All that day the fever raged. The sufferer lay stretched full length on his bed, staring at vacancy, talking in a low tone incoherently to himself. Every now and then a tremor seized his hands, every now and then they picked convulsively at the bed-clothes. The muscles of his face twitched frequently, giving it a grim aspect. His breathing was quick and shallow. Thus he continued throughout the day, with little change, until Grant came again in the evening.

All that day, after some few hours' rest, Sister Olive sat and tended him with a care and solicitude that could not be surpassed. Every alternate hour her hand administered those stimulants that Dr. Grant had ordered, to supply the ravages made in that terrible and exhausting conflict going on within. At intervals the sufferer would fall into uneasy sleep, then again became wakeful and delirious. Mrs. Tenison was almost beside herself with grief, the more terrible that it was pent-up. And it was not time to buoy her up with false hopes. She knew as well as Dr. Grant that it was a struggle for life.

And so through the long night again the delirium raged. But not a jot did the two relax their efforts. Sister Olive went to her room at midnight. Dr. Grant, satisfied from various signs that the climax was approaching, sat up. And a weary watch it was.

There had been no change for some hours when Grant left in the morning. But, soon after he had left, Olive and the mother, watching still, saw their patient fall into a profound sleep.

When Dr. Grant called at mid-day, he was still sleeping and the temperature was lower. He ventured to say to Mrs. Tenison:

"It is a better sign. I do not say all danger is over yet, but there is less danger."

The mother could say little: but she plucked up heart, and Sister Olive encouraged her. Still the women watched. At last the moment came. The sleep ended. The temperature had gradually gone down to nearly its normal level, and when the regular breathing stopped, and the patient stirred, then turned his head slightly, looked around, as if half-dazed, and said in a weak voice, "Mother!" the pent-up tears welled forth, and Sister Olive glided from the room to leave mother and son together.

That afternoon Archie was alone with Sister Olive. He was lying very comfortably. Soothing fomentations had been applied to his wounded skin. There was little pain, only he felt wonderfully weak. After some pause he said:

"Sister Olive! You've been awfully good to me all through this. I don't think I could have pulled through if it hadn't been for Grant and you. I've been wondering how it was Grant came; and I think it must have been through you. You sent for him, didn't you?"

"Yes, Archie! I did—and I'm glad I did."

"Well, you know, I didn't like Grant before. But now, from what my mother's told me, I see I've been making a great mistake. I looked on Grant as a rival, and fancied he meant to slight me on purpose, when it was only my morbid sensitiveness. But now I know a little better what a grand fellow he is. He's much more worthy of you than I am, and I'll be quite satisfied if you always remain to me like a real sister. You know I never had one; and you've been such a sister to me through this illness, I don't quite know how I can ever . . ."

"Archie!" Sister Olive interrupted, "you really must not talk so much just now, and weary yourself. We'll have a long talk when you get better. There's a ring at the bell!"

"Ah! that's Grant," said Archie. "I say, Sister Olive; you're not offended at what I said just now?"

"Offended! no!" she said. "But we won't talk of it any more, just now."

Presently Dr. Grant entered. A great look of thankfulness came to his face when he saw how matters stood, and Sister Olive went off to have a chat with Mrs. Tenison.

"I suppose the worst is over now, Grant?" said Archie, after a few words had passed. "And I can't express how much I feel I owe this recovery to you and Sister Olive. I've been trying to say something of it to her, but"—and here a slight smile came to his lips—"it was a sort of failure, and I guess this is going to be another. Grant, perhaps you won't credit it, but I used to have pretty hard thoughts of you before this, and now I want to confess that I was quite wrong, and didn't allow myself to appreciate you as you deserved. Will you forgive me?" and he held out his hand.

"Archie!" said Dr. Grant, in a low tone, grasping the hand warmly. "Say no more about it. Men don't always learn to appreciate one another till some trouble comes, and that teaches them. Henceforth, we shall be firm friends, and if I can help you at any time, I'll do it. Only don't stand aloof. Just come."

"There's another thing on my mind, Grant. I think I can see how things are between you and Sister Olive. I was a little sore in that quarter once. But that's past now. I love her as much as ever, but not in that way. I hope you'll win her."

"Archie!" said Dr. Grant, "if you've guessed a secret, keep it. When there's any definite news, I'll tell you. I'm very glad that you've been spared to get through this struggle, and pleased beyond anything that we have come to know one another better."

Later on that evening, Sister Olive and Doctor Grant were sitting by the fire downstairs, while Mrs. Tenison was in the bedroom with her boy. Grant once more approached the subject that lay nearest to his heart. They had been together to see John Hargen, at the close of the afternoon.

"Now, Sister Olive," he began, "what are you going to do? Are you going back to hospital work?"

"I don't quite know, Dr. Grant," said Sister Olive. "I must wait till John is quite well. I did think that, if he got an appointment in London, I would go and keep house for him. But he doesn't seem to think with me. He says he must work out his own salvation alone, and won't have me with him till he has made a home to offer me. Perhaps it will be well to take him at his word, and let it be so. But before he begins work he is going home with me. Time has softened my father and mother a little. I think, at last, they begin to see through Lumley. Perhaps my father will get John another appointment."

"And after that?" Dr. Grant asked with emotion, taking her by the hand. "Olive, will you let me find a new home for you? There are no secret clouds between us now. I am ready to take all the possibilities. Your brother, I am assured, will never cause you a single pang again. Will you take charge of me, Olive?"

Grant had some news for Archie that night, and Archie remarked, "I must make haste and get well, or I shan't be in time for that wedding, or be able to propose the health of 'Sister Olive.'"

